# Long-Term Comparative Outcomes of Short Implants Versus Maxillary Sinus Elevation in Posterior Maxilla Rehabilitation

**DOI:** 10.3390/dj13010012

**Published:** 2024-12-27

**Authors:** Eduardo Anitua, Laura Piñas, Mohammad Hamdan Alkhraisat

**Affiliations:** 1 University Institute for Regenerative Medicine and Oral Implantology—UIRMI (UPV/EHU-Fundación Eduardo Anitua), 01007 Vitoria, Spain; lapica77@gmail.com (L.P.); mohammad.hamdan@bti-implant.es (M.H.A.); 2 BTI Biotechnology Institute, 01005 Vitoria, Spain; 3 Eduardo Anitua Dental Clinic, 01007 Vitoria, Spain; 4 Oral and Maxillofacial Surgery, Oral Medicine and Periodontics Department, Faculty of Dentistry, University of Jordan, Amman 11942, Jordan

**Keywords:** short dental implant, sinus lift, maxillary bone atrophy

## Abstract

**Background**: Vertical atrophy of the maxilla has traditionally been treated with sinus lift procedures and implant placement, performed in one or two surgical stages. Subsequently, the transcrestal sinus lift technique was introduced, offering distinct advantages in terms of indications and reduced morbidity. Most recently, short implants have emerged as a valid alternative to these procedures, even in cases of severe horizontal resorption, allowing for direct placement in many cases. This study was designed to assess the clinical outcomes of short implant placement in alveolar ridges with severe bone atrophy, compared with conventional-length implants placed in areas undergoing conventional sinus elevation. **Methods**: A retrospective split-mouth study was conducted to compare conventional sinus elevation with standard-length implants versus short implants for addressing vertical bone atrophy in the posterior maxilla. The primary variable was the variation in the marginal bone level. The secondary variables were implant survival and complications. The evaluation of the statistical significance of the difference in categorical variables was accomplished by Chi-squared test or Fisher’s exact test. The comparison between the study groups in continuous variables was performed using Wilcoxon test. The statistical significance was set at *p*-value < 0.05. **Results**: The study sample consisted of 24 patients and a total of 73 dental implants. The lateral sinus elevation group (LSEG) included 39 implants, while the short implants group (SIG) included 32 implants. All prostheses were screw-retained. Changes in marginal bone levels indicated a marginal bone loss of less than 0.5 mm in both groups, with no statistically significant difference. In the LSEG, two cases of mucositis were identified, attributed to improper use of an interdental brush. Additionally, two cases of prosthetic screw fracture were reported in the LSEG as technical complications. **Conclusions**: Long-term outcome data have provided evidence that the use of short implants is comparable to a state-of-the-art procedure (sinus grafting and placement of implants) regarding implant survival, marginal bone remodeling, and complication rates.

## 1. Introduction

The global population is undergoing a significant demographic shift, with an unprecedented rise in the number of elderly individuals [1,2]. This demographic change has increased the demand for dental implant treatments among older patients, who often seek these solutions to improve their quality of life [3,4]. Aging is frequently linked to progressive alveolar bone atrophy, which is especially characterized by reductions in bone height and width, complicating standard implant placement [5]. This phenomenon primarily arises from alveolar ridge resorption following tooth loss, a chronic, irreversible, and cumulative process [6]. A significant portion of bone loss occurs within the first year following tooth loss, with the highest rate observed during the initial months [7]. Research has highlighted that the mandible and posterior regions are more susceptible to resorption compared to the maxilla and anterior regions, respectively. Studies on cadaveric skulls from the 7th–8th century A.D. revealed a predominance of knife-edge patterns in the anterior alveolar ridges of both jaws, while posterior segments often exhibited rounded ridges with reduced height or depressions in bone level [8]. Tooth loss has also been associated with decreased bite force and reduced activity of masticatory muscles, particularly in denture-wearing individuals [9]. This hypofunction contributes to alterations in bone composition, including reduced mineralization and diminished collagen content. These changes, coupled with an increased prevalence of over-aged bone (fewer viable osteocytes), undermine the stability of the alveolar bone and elevate the risk of resorption [10,11,12]. Other pathological factors like periodontal diseases, trauma, and surgical interventions increase the risk of bone loss, and would further limit the alveolar bone volume.

In response, implantology has evolved to address these specific anatomical and clinical challenges [5]. Traditional surgical interventions—such as sinus lifts, block grafting, and guided bone regeneration—have been long-standing solutions for managing severe vertical bone resorption [13,14,15,16,17,18,19]. However, the associated costs and procedural morbidity have catalyzed the search for alternative approaches that are both predictable and minimally invasive [3,4]. This shift led to the development of short implants, defined as implants under 8 mm in length, which offer a less invasive alternative to vertical bone augmentation surgeries. Long-term data have shown that short implants demonstrate predictable clinical performance (survival and remodeling of the marginal bone), comparable to longer implants [20,21,22]. When comparing outcomes for short implants with traditional bone augmentation and longer implants in the maxilla, no significant differences were found in survival rates or bone stability, though short implants showed fewer biological complications [21,22,23,24]. Similar findings have emerged in the mandible, where short implants not only reduce post-surgical complications and bone loss after five years of follow-up, but also eliminate the need for complex inferior alveolar nerve repositioning procedures [24,25,26,27]. A recent systematic review, encompassing 1269 patients and 2631 implants across 1460 studies, found statistically similar outcomes in survival rates, bone remodeling, or biological and mechanical complications when comparing short implants with longer ones [28]. Thus, these innovations not only simplify the surgical process, but also provide patients with safer, more reliable pathways to functional restoration, reflecting the trend toward minimally invasive techniques in dental implantology [29,30].

Although the clinical data of short implants are promising, their application in alveolar ridges with severe bone atrophy still warrants further investigation to confirm their long-term efficacy and safety. The primary challenge lies in the reduced bone height in atrophic ridges, which can negatively affect the primary stability of short implants. Limited primary stability is a known risk factor for inadequate osseointegration, as insufficient bone volume may lead to micromovements that compromise the integration of the implant into the surrounding bone matrix [31]. Moreover, in the maxilla, extreme bone atrophy increases the probability of implant loss into the maxillary sinus, a potential complication that can occur when implants are placed in areas with less than 4 mm of bone height [32,33,34]. Studies suggest that achieving primary stability in cases with minimal vertical dimension of the alveolar bone often requires careful consideration of implant design, surface modifications, and insertion techniques to enhance initial retention and improve osseointegration potential [35,36,37,38,39]. Although recent advances in implant surface technology and modified surgical protocols have shown improved outcomes in challenging cases, continued research is necessary to establish standardized protocols for short implants in severely atrophic ridges.

Several studies have explored the use of short implants in patients with severe alveolar bone atrophy in the posterior maxilla [40,41,42]. Carelli et al. combined transcrestal sinus elevation (up to 8 mm) with bone grafting in cases with residual bone height <3 mm. After 5 years of follow-up, none of the 30 short implants have failed, showing a marginal bone loss of −0.33 ± 0.11 mm [40]. Similarly, Amato et al. placed 20 short implants with transcrestal sinus elevation without grafting in cases with residual alveolar bone height less than 5 mm, also reporting no implant failures [41]. Chen et al. placed 25 short implants simultaneous to transcrestal sinus floor elevation, reporting that none of the implants failed [42]. Despite these promising results, there is a need for comparative clinical studies to overcome the shortage in long-term outcome data to support the shift from the state-of-the-art procedure (sinus grafting and placement of standard implants) to a less invasive approach using short implants. To address this gap, the present study has been performed to evaluate the clinical outcomes of short implant placement in alveolar ridges with severe bone atrophy (≤3 mm), compared with conventional-length implants placed in areas undergoing conventional sinus elevation. Designed as a split-mouth study, this study enables direct comparison of both procedures within the same patient under identical conditions. The working hypothesis is that the use of short implants in patients with atrophic posterior maxilla will maintain the radiographic marginal bone stability without adverse effects.

## 2. Materials and Methods

This retrospective study was performed in agreement with the guidelines of the Helsinki declaration and its subsequent amendments regarding human clinical research. This study was approved by the local ethical committee (FIBEA-01-ER-24-Atrofia severa). All patients were previously treated as part of the routine clinical practice, by the same clinicians, employing the same implant system and the same surgical protocols. The study groups were the lateral maxillary sinus elevation group (LSEG) and the short implants group (SIG).

After the study approval, a pseudonymized database was created. The study sample consisted of male and female patients over 18 years of age who had lateral maxillary sinus elevation on one side and extra-short implants (≤6.5 mm in length) on the contralateral side. Patients were excluded if a radiographic image at the time of loading was unavailable.

The main variable was the variation in the marginal bone level. The secondary variables were implant survival and complications. For that, demographic (sex and age), surgery, and implant-related variables, as well other prosthetic-biomechanical variables were obtained from patients’ records.

### 2.1. Implant Placement—Surgical Protocol

Patient implant surgery was performed after receiving periodontal treatment. Treatment planning was decided considering clinical data, wax-up of the case, and radiographic examination. Cone-beam computed tomography (CBCT) scans were used to assess the alveolar bone and associated anatomical structures using specialized software for implant surgery planning (BTI Scan; BTI Biotechnology Institute, Vitoria, Spain).

In the LSEG, lateral maxillary sinus elevation was performed by opening a lateral window in the mesial wall of the maxillary sinus using piezoelectric surgery. The Schneiderian membrane was detached and elevated to create space for the bone graft. Anorganic bovine bone (Bio-Oss, Geistlich Pharma, Wolhusen, Switzerland) mixed with Plasma rich in Growth Factors (PRGF; KMU 15; BTI Biotechnology Institute, Vitoria, Spain) in 2:1 (*w*/*v*) ratio was grafted beneath the Schneiderian membrane. The lateral window was then repositioned and covered with a PRGF membrane before flap closure. Implant surgery was performed 4–6 months later.

For both groups (LSEG and SIG), the same surgeon (EA) prepared the implant site. For that, biological drilling at low speed (150 rpm) without irrigation was performed. The diameter of the final bone drill was selected based on the bone type. In the SIG, transcrestal sinus elevation was performed by using the same surgical drills that were used to prepare the implant site. A frontal cutting drill was used to negotiate the basal cortical bone and a clot of PRGF was then introduced to push the Schneiderian membrane. Before implant placement, the implant sites were irrigated with PRGF. For implant loading, transepithelial abutments (BTI Biotechnology Institute, Vitoria, Spain) were used in all cases. Impression copings for the intermediate Multi-Im^®^ abutments and polyether impression material (Impregum Penta, 3M ESPE, Maplewood, MN, USA) were used to make an impression using the open-tray technique. The occlusal scheme was maximum intercuspation and centric relation for partially and completely edentulous cases, respectively.

**Figure 1:**
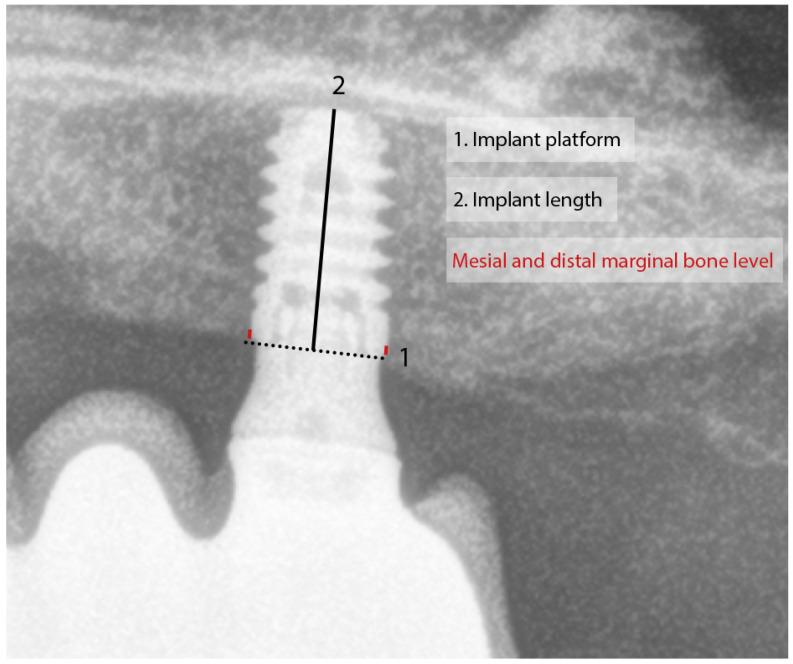
Measurement of the radiographic marginal bone level. The known length of the implant was used for measurement calibration. Mesial and distal marginal bone level was measured from the implant platform to the most coronal point of implant–bone contact.

The postsurgical assessment included clinical evaluation of the implant and prosthesis and radiographic assessment of the implant–bone interface. Implant survival was defined by the presence of the dental implant in the alveolar bone at the last follow-up. Changes in marginal bone level were assessed by measuring the distance between the implant’s prosthetic platform and the most coronal point of implant–bone contact. The radiographs were calibrated by the implant length (Figure 1). Measurements were taken mesially and distally to the implants, and they were repeated in the radiograph at implant loading and the last available radiograph.

### 2.2. Statistical Analysis

The statistical analysis was conducted in specialized software [SPSS Statistics version 15 (IBM, Armonk, NY, USA)]. Absolute and relative frequencies were used to describe categorical variables. The normal distribution of continuous variables was assessed using the Shapiro–Wilk normality test. Continuous variables were described by the median and the range. The evaluation of the statistical significance of the difference in categorical variables was accomplished by the Chi-squared test or Fisher’s exact test. The comparison between the study groups in continuous variables was performed using Wilcoxon test. The statistical significance was set at *p*-value < 0.05.

## 3. Results

The study sample had 24 patients and 71 dental implants; 16 patients were females and 8 were males. The patients’ mean age was 58 ± 9 years (range: 38 to 76 years).

**Figure 2:**
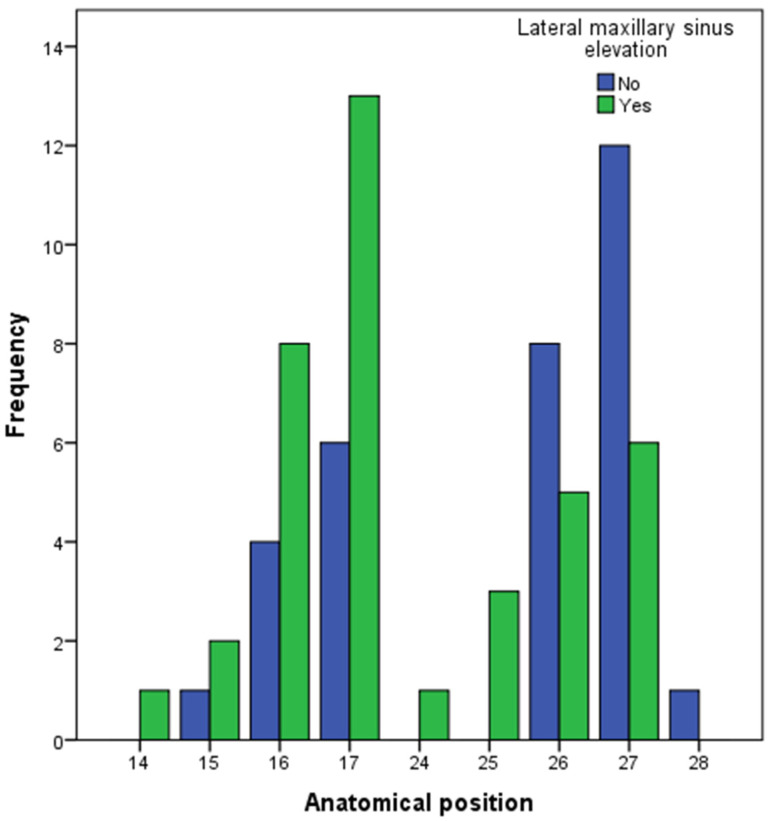
Anatomical position of the dental implant included in this study. Implant position was defined following the FDI tooth numbering system.

The lateral sinus elevation group (LSEG) had 39 implants and the short implants group (SIG) had 32 implants. All of the implants were placed in the premolar–molar region of the maxilla (Figure 2). The differences in the implant position were not statistically significant between the study groups (Chi-squared test; *p*-value: 0.136).

**Figure 3:**
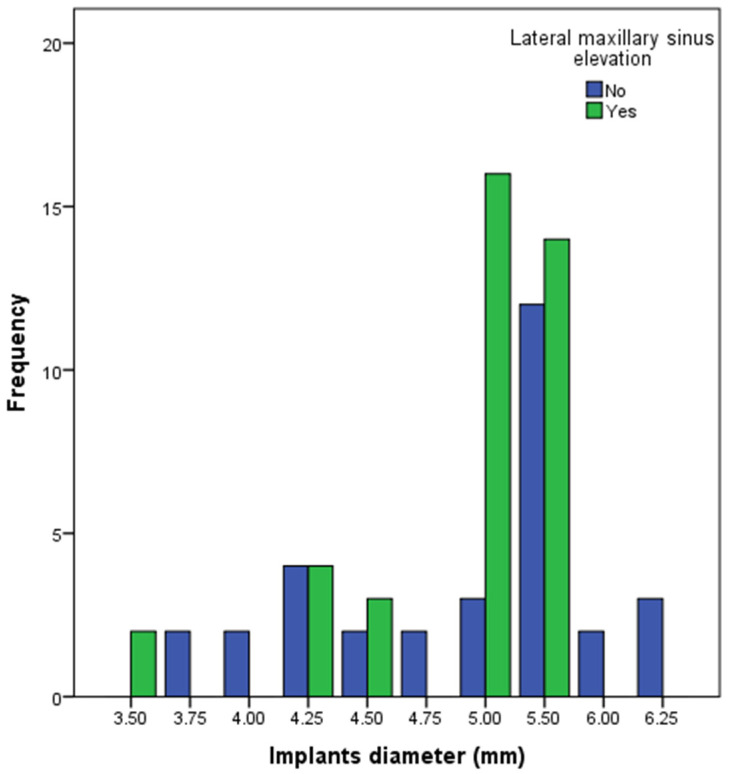
The diameter of the dental implants included in this study.

**Figure 4:**
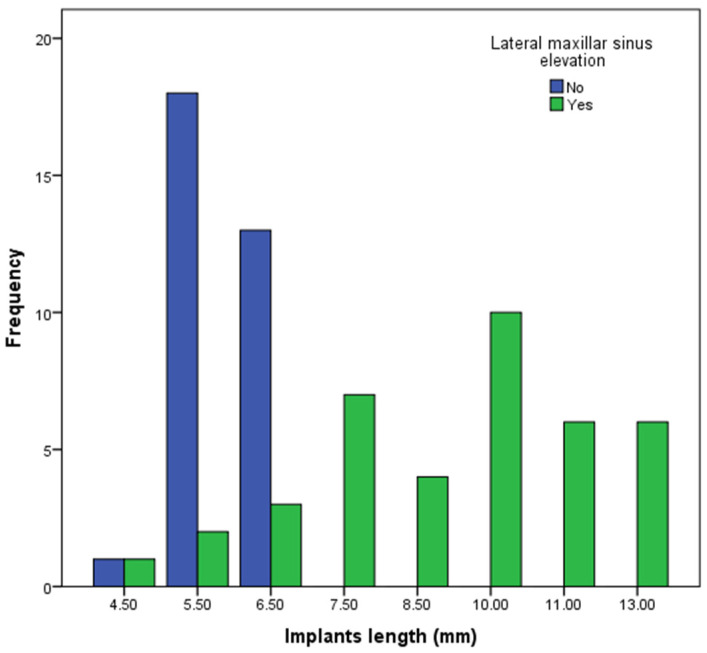
The length of the dental implants included in this study.

Figure 3 and Figure 4 show the diameter and lengths of the dental implants. The diameters 5.0 and 5.5 mm comprised 30 implants (76.9%) in the LSEG. However, a wider distribution of the implant diameters was observed in the SIG. The differences in the frequency of the implant diameters were statistically significant (Chi-squared test; *p*-value: 0.010). The length ≥10.0 mm comprised 22 (56%) implants in the LSEG; 6 implants were ≤6.5 mm in length. All of the implants in the SIG were ≤6.5 mm in length. The differences between the two groups in the frequency of the implant lengths were statistically significant (Chi-squared test; *p*-value: 0.000).

**Figure 5:**
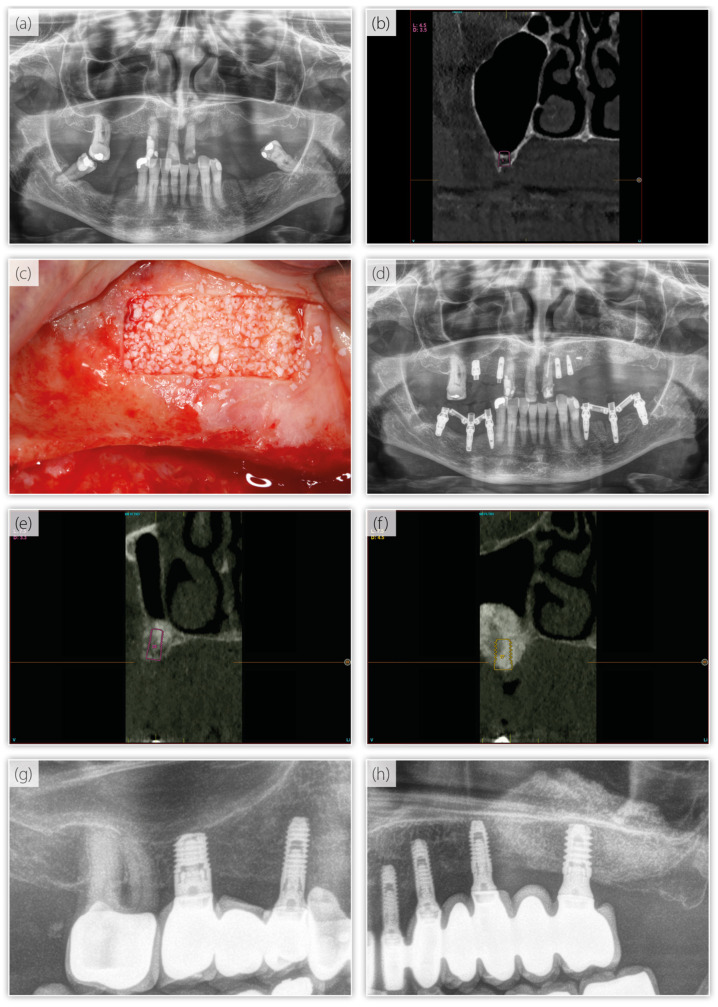
(**a**) Panoramic radiograph at the baseline. (**b**) Implant planning for the first quadrant, featuring a short implant to be placed directly. (**c**) Image of the lateral maxillary sinus elevation in the second quadrant. (**d**) Radiograph after the placement of the dental implants. (**e**,**f**) Implant planning in the sinus elevation area following graft integration. (**g**,**h**) Good clinical performance of all the implants.

Table 1 shows that most of the implants were inserted to give support to bridges in comparison with other types of prostheses. All of the prostheses were screw-retained. The antagonist type was implant-supported prothesis in most of the cases. There were no statistically significant differences between the groups in these two variables. The crown-to-implant ratio was significantly higher in the SIG. The patients were followed for a median of 108 months (range: 8 to 208 months) (Table 2); 75% of the cases had a follow-up time ≥5 years. During this time, one implant belonging to the SIG group failed, being not statistically significant. The bone density before implant placement showed the absence of statistically significant differences between the study groups (Table 3). The variation in the marginal bone level indicated marginal bone loss of <0.5 mm in both groups, also being not statistically significant (Table 3). Two cases of mucositis, due to the improper use of the interdental brush, were identified in the LSEG. Regarding technical complications, two cases of prosthetic screw fracture were identified in the LSEG (Table 1). Figure 5 illustrates a clinical case included in this study.

## 4. Discussion

The physiological phenomena of maxillary sinus pneumatization and alveolar ridge remodeling would limit the height of the residual alveolar bone in the posterior maxilla. This would often require maxillary sinus floor augmentation to achieve sufficient bone volume for the insertion of dental implants.

Traditionally, lateral sinus floor elevation (SFE) has been widely used to treat atrophic posterior maxilla, allowing placement of standard-length implants [16]. The lateral SFE involves the opening of a lateral window into the maxillary sinus, access to and elevate the Schneiderian membrane, and add grafting material beneath. A healing time of 4 to 6 months is often needed. Studies have shown that this technique can achieve high success rates for implant survival and stability, particularly when bone grafts effectively integrate with the host bone [43,44,45]. However, lateral SFE procedures can involve increased patient morbidity, higher costs, and extended treatment times [43]. Additionally, complications such as membrane perforation, graft infection, and postoperative discomfort are relatively common [46,47]. Another less invasive approach, the transcrestal sinus floor elevation, has been first reported by Tatum, where the maxillary sinus is accessed through the alveolar bone crest [48,49]. Compared to lateral SFE, the transcrestal approach offers the advantages of short rehabilitation time and cost-effectiveness [48,49]. The simultaneous implant placements with sinus floor elevation requires sufficient implant primary stability to lower the risk of implant migration into the sinus and osseointegration failure [37,50]. Several strategies have been described to enhance the implant primary stability such as the use of wider implants, adapt site under-preparation to the quality of alveolar bone, and the implant design (roughened surface, aggressive threads, and/or tapered designs) [37]. Consequently, an alternative approach—using short implants—has gained interest for its minimally invasive nature, which may reduce complications and treatment time while providing similar outcomes [24,29,31]. These implants are designed to engage the limited available bone without additional bone grafting. Research supports that short implants can achieve survival rates comparable to those of standard implants in SFE cases, with some studies reporting comparable marginal bone loss and implant stability rates, but consistently fewer complications with short implants [21,47,48,49].

The shift from the state-of-the-art procedure (sinus grafting and placement of standard implants) to a more modern, less invasive approach using short implants is constrained by the limited availability of long-term outcome data. This study provides long-term comparative data of the two approaches in a split-mouth scheme (the procedures performed in the same patient).

In this study, we did not observe statistically significant differences between the two techniques regarding marginal bone loss, implant failure, or the incidence of peri-implant disease (peri-implant mucositis). These findings indicate that both procedures yield similar results across the analyzed parameters. Our results align with long-term data (10 years) from two randomized clinical trials examining short (≤6 mm) implants.

Thoma et al. [51] evaluated 32 patients with 47 long implants and 29 patients with 39 short implants, reporting survival rates of 100% and 97% for the long and short implants, respectively. Both groups have shown almost stable marginal bone level (MBL) of 0.00 mm, ranging from 0.00 to 4.55 mm for long implants and 0.00 to 3.25 mm for the short implants. In contrast, Guljé et al. [52] studied 9 patients with 10 long implants and 6 patients with 17 short implants, reporting slightly lower survival rates of 90.9% and 89.5% for long and short implants, respectively. The average MBL for long implants has been 0.26 mm ± 0.12 mm, while short implants have shown an average MBL of 0.18 mm ± 0.10.

In our study, the mean marginal bone level (calculated from the average mesial and distal bone loss in the patients studied) in our case was 0.2 mm at loading for both groups—short implants and sinus lift. In the most recent radiograph, the marginal bone level was 0.75 mm for the lateral sinus elevation group and 0.55 mm for short implants group, with no statistically significant differences observed between them.

Similar results are observed in other studies comparing these treatment groups, such as Felice et al. [53], reporting 0.55 mm for short implants and 0.61 mm for conventional-length implants, and Hi et al. [54], with 0.51 mm for short implants and 0.52 mm for conventional implants, although in this latter study, the procedure for placing longer implants involved transcrestal sinus elevation. Other studies, however, report higher bone loss for both groups, such as Esposito et al. [55], showing 1.30 mm for short implants and 1.48 mm for long implants, and Pistilli et al. [56], reporting 1.33 mm for short implants and 1.44 mm for conventional-length implants.

However, notable distinctions between the techniques regarding implant length and diameter have been observed. Implants in conventional sinus elevation areas tend to be significantly longer, whereas short implants generally have a wider diameter [51,57]. This variation can be attributed to the evolution of both techniques and the continuous search for optimal anchorage. In earlier years, transcrestal sinus elevation aimed to achieve primary stability through implant length, leading to the placement of the longest possible implants in the regenerated bone [56]. Currently, with the use of short implants and a progressively more conservative approach in dentistry, the goal has shifted toward achieving three-dimensional stability by maximizing contact across the entire ridge surface [49]. The approach with short implants is more effective, particularly for enhancing bone stability in low-density areas like the atrophic posterior maxilla [49,50,58]. The need to stabilize an implant in a bed with limited residual bone volume may be the reason why some studies, such as the meta-analysis conducted by Bitinas et al. in 2021 [59], have observed a higher failure rate of implants. This, combined with the heterogeneity of studies that evaluate different implant insertion techniques and various types of short implants with different morphologies, may contribute to the lower survival rates observed for these implants [59,60,61]. In our study, short implants were placed using a specific drilling protocol that assesses the implant bed prior to placement, adjusting the drilling approach based on bone quantity and quality [37]. Furthermore, the short implant group is heterogeneous in size, with lengths consistently under 8 mm, making the results more comparable.

The use of short implants in the reconstruction of the posterior maxilla has emerged as a promising approach, with increasing support from long-term outcome data. Our results agree with other studies that have investigated the application of short implants in patients experiencing severe alveolar bone atrophy in the posterior maxilla [40,41,42]. In the study by Carelli et al., all 30 short implants remained successful after 5 years of follow-up, with a marginal bone loss averaging −0.33 ± 0.11 mm [40]. Likewise, Amato et al. placed 20 short implants in atrophic posterior maxilla, reporting zero implant failures [41]. Chen et al. also reported no implant failures among the 25 short implants that were placed in the posterior maxilla [42].

This study contributes to the growing body of evidence by providing data derived from a split-mouth design, which was employed to minimize biological variability and enhance the reliability of the results. By utilizing this design, we were able to directly compare the outcomes of both techniques (sinus lift and short implants) within the same patient, offering a more controlled assessment of their relative performance. Furthermore, it provided outcome data from long-term follow-up.

However, despite its strengths, this study is not without limitations. The retrospective design introduces inherent biases, as it relies on the analysis of pre-existing data, which may not always have been collected in a standardized manner. Additionally, the dependency on database availability limits the scope of data, as certain relevant information may be missing or incomplete. These factors should be considered when interpreting the findings, as they could influence the generalizability of the results. This study was not designed to assess the influence of systemic diseases on the outcomes of both techniques in the treatment of atrophic posterior maxilla, opening the door for future studies to bridge this gap.

## 5. Conclusions

Long-term outcome data have provided evidence that the use of short implants is comparable to a state-of-the-art procedure (sinus grafting and placement of implants) in terms of implant survival, marginal bone loss, and complication rates. Clinicians may reconsider the use of the maxillary sinus elevation procedure and opt for short implants in the prosthetic rehabilitation of posterior maxilla. Future research is required to obtain long-term data coming from prospective clinical trials and to assess the influence of confounding factors (systematic diseases, anatomical variations, among others) on these outcomes.

## Figures and Tables

**Table 1:** Prosthesis-related variables.

Variable		Lateral Sinus Elevation Group	Short Implants Group	*p*-Value
Prosthesis type for each implant	Crown	0	1	0.527 ^a^
	Bridge	31	24	
	Complete denture	8	7	
Crown-to-implant ratio		1.3 (0.8 to 4.4)	2.2 (1.0 to 3.0)	0.000 ^b^
Antagonist type for each implant	Implant-supported	30	25	0.916 ^c^
	Tooth/tooth-supported	7	6	
Prosthetic screw fracture		2	0	0.498 ^c^

^a^ Chi-squared test; ^b^ Wilcoxon test; ^c^ Fisher’s exact test.

**Table 2:** Follow-up time of the implants included in this study.

Follow-Up Time (Months)	Implants
8	1
11	1
20	1
21	4
24	1
27	1
32	1
43	1
44	2
47	1
49	1
51	2
53	1
59	4
62	1
71	1
72	1
79	1
83	1
91	1
93	1
98	1
99	1
102	1
104	1
105	2
108	1
109	1
121	1
122	2
126	2
130	2
134	2
140	1
144	2
156	2
163	2
169	1
171	2
172	1
174	3
175	1
177	1
180	1
181	3
204	1
206	2
208	2

**Table 3:** Dental implant-related variables.

Variable		Lateral Sinus Elevation Group	Short Implants Group	*p*-Value
Number of implants		39	32	
Bone density (median; range)		700 (300–1100)	450 (100–700)	0.071 ^a^
Mesial MBL ^b^ (mm) (median; range)	loading	0.0 (−1.6 to 0.3)	0.0 (−0.71 to 0.0)	0.088 ^a^
Distal MBL ^b^ (mm) (median; range)		−0.4 (−2.2 to 0.0)	−0.4 (−0.8 to 0.0)	0.789 ^a^
Mesial MBL ^b^ (mm) (median; range)	Last radiograph	0.7 (−3.9 to 0.0)	0.4 (−1.0 to 0.0)	0.005 ^a^
Distal MBL ^b^ (mm) (median; range)		−0.8 (−3.2 to 0.4)	−0.7 (−3.1 to 0.0)	0.155 ^a^
Variation in MBL ^b^–mesial (mm) (median; range)		−0.3 (−3.9 to 0.1)	−0.2 (−1.0 to 0.1)	0.092 ^a^
Variation in MBL ^b^–distal (mm) (median; range)		−0.4 (−3.2 to 0.8)	−0.2 (−2.8 to 0.32)	0.196 ^a^
Implant failure (frequency)		0	1	0.45 ^c^
Mucositis		2	0	0.498 ^c^

^a^ Wilcoxon test; ^b^ MBL: marginal bone level; ^c^ Fisher’s exact test.

## Data Availability

The datasets used and/or analyzed during the current study are available from the corresponding author on reasonable request.
